# [Corrigendum] Sensitizing TRAIL-resistant A549 lung cancer cells and enhancing TRAIL-induced apoptosis with the antidepressant amitriptyline

**DOI:** 10.3892/or.2025.8920

**Published:** 2025-05-29

**Authors:** K.M.A. Zinnah, Sang-Youel Park

Oncol Rep 46: 144, 2021; DOI: 10.3892/or.2021.8095

Subsequently to the publication of the above paper, an interested reader drew to the authors' attention that, for the cellular morphological images shown in Figs. 3A and 4A on p. 7 and 8 respectively, the centrally placed images (third from the left) were strikingly similar, such that the same data had apparently been included in these figures to show the results from differently performed experiments.

After having re-examined their original data files, the authors realized that the data panel in Fig. 4A properly belonged to Fig. 3, and that this image had been inadvertently included incorrectly in this figure. The revised version of Fig. 4, now featuring the correct data for the ‘Amit+/TRAIL+/DR5 siRNA-/NC -’ experiment (third panel from the left), is shown on the next page. Note that the correction of this figure does not affect the overall conclusions reported in the paper. The authors are grateful to the Editor of *Oncology Reports* for allowing them the opportunity to publish this Corrigendum, and apologize to the readership for any inconvenience caused.

## Figures and Tables

**Figure 4.**
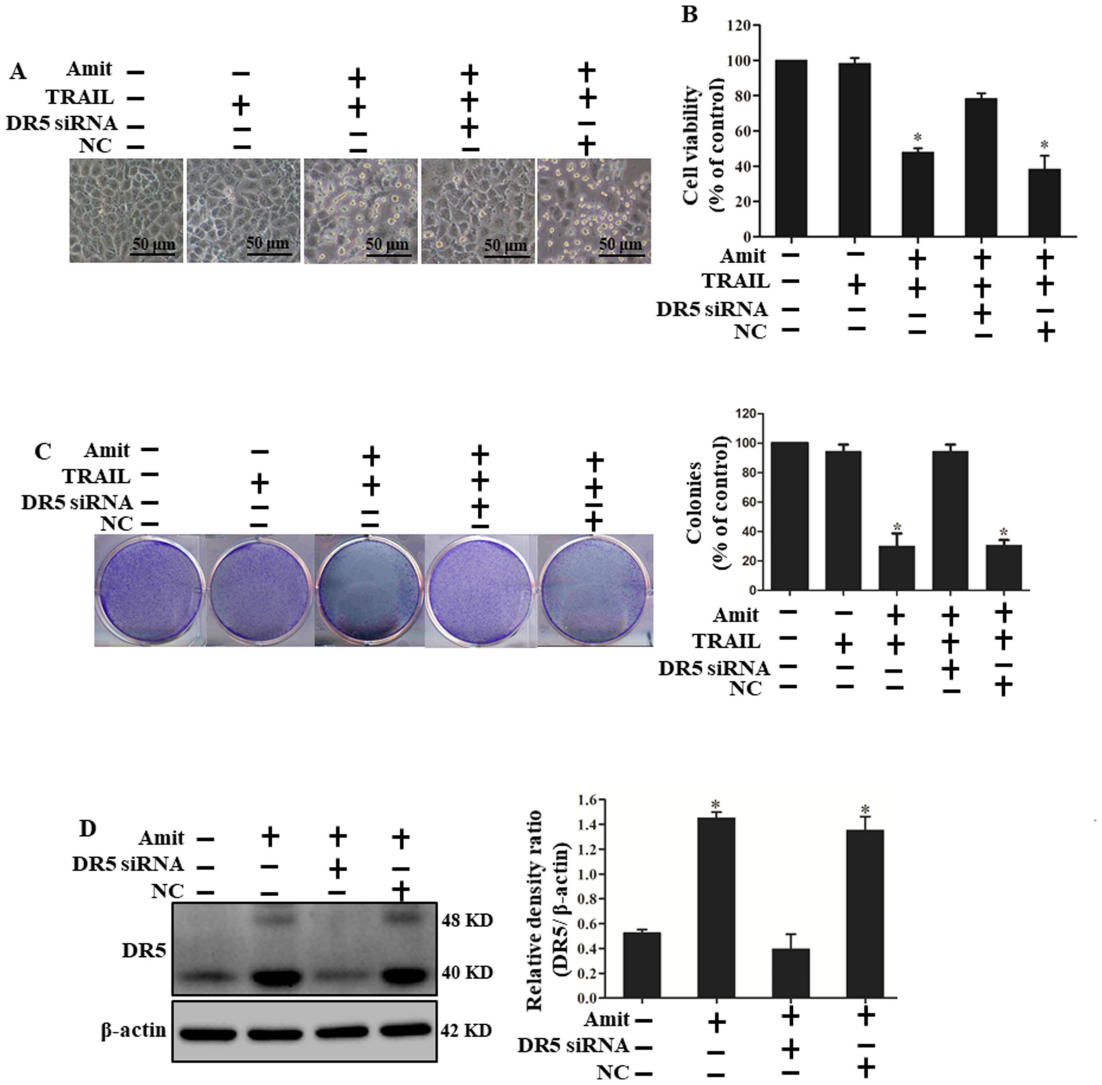
Silencing of DR5 expression negatively controls amitriptyline-induced TRAIL-mediated apoptosis. DR5 siRNA and control siRNA (40 nM) were transfected for 24 h, then the cells were treated with amitriptyline (40 µM) for 12 h and finally, 100 ng/ml of TRAIL protein was added for 3 h. (A) Images of the cells were captured and morphological variations were examined under a light microscope (magnification, ×100; scale bar, 50 µm). (B) Cell colonies were stained with crystal violet dye and the number of colonies were counted. (C) MTT assays were performed to reveal the cell viability percentages (bar graph). Statistically significant differences between the control and each indicated treatment group are presented as *P<0.001. (D) Whole-cell lysates were prepared and analyzed by western blotting to determine the expression of DR5. TRAIL, tumor necrosis factor-related apoptosis-inducing ligand; DR, death receptor; siRNA, small interfering RNA; NC, negative control; Amit, amitriptyline.

